# Supplementary material for: Getting More Out of Biomedical Documents with GATE's Full Lifecycle Open Source Text Analytics
Source: PLoS Comput Biol. 2013 Feb 7;9(2):e1002854. doi: 10.1371/journal.pcbi.1002854 (PMC3567135; doi:10.1371/journal.pcbi.1002854)
Supplement: Dataset S1 — GATE software. Dataset S1 bundled with this paper contains a distribution of GATE (or see http://gate.ac.uk/download/). (TGZ) [file pcbi.1002854.s001.tgz › plos-gate/plugins/Parser_SUPPLE/docs/manual.pdf]

SUPPLE:
The Sheffield University Prolog Parser for Language Engineering

Horacio Saggion and Mark A. Greenwood
Department of Computer Science
University of Sheffield

September 30, 2005

A royalty-free license is granted for the use of this software for NON_COMMERCIAL PURPOSES ONLY.

The program is provided “as is” without warranty of any kind, either expressed or implied, including, but not limited to, the implied warranties of merchantability and fitness for a particular purpose. The entire risk as to the quality and performance of the program is with you. Should the program prove defective, you assume the cost of all necessary servicing, repair or correction.

Chapter 1

Introduction

SUPPLE is a bottom-up parser that constructs syntax trees and logical forms for English sentences. The parser is complete in the sense that every analysis licensed by the grammar is produced. In the current version only the 'best' parser is selected at the end of the parsing process. The English grammar (see Chapter 8) is implemented as an attribute-value context free grammar which consists of subgrammars for noun phrases (NP), verb phrases (VP), prepositional phrases (PP), relative phrases (R) and sentences (S). The semantics associated with each grammar rule allow the parser to produce logical forms composed of unary predicates to denote entities and events (e.g., *chase(e1), run(e2)*) and binary predicates for properties (e.g. *lsubj(e1,e2)*). Constants (e.g., $e1$, $e2$) are used to represent entity and event identifiers. The Gate SUPPLE Wrapper stores syntactic infomation produced by the parser in the gate document in the form of: SyntaxTreeNodes which are used to display the parsing tree when the sentence is 'edited'; 'parse' annotations containing a bracketed representation of the parse; and 'semantics' annotations that contains the logical forms produced by the parser.

Chapter 2

Software requirements

In order to compile and use the parser you will need the following software:

- SUPPLE parser and Gate wrapper
  Available from `http://nlp.shef.ac.uk/research/supple/`
- The GATE Framework
  Available from `http://gate.ac.uk`
- Java (v1.4 or above) Available from `http://www.javasoft.com`
- Ant (to construct the parser)
  Available from `http://ant.apache.org`
- A Prolog implementation. We currently support:
  - SICStus prolog (v3.8.6 or above)
    For details see `http://www.sics.se/sicstus/`
  - PrologCafe (v0.9 or above)
    Available from `http://kaminari.scitec.kobe-u.ac.jp/PrologCafe/`
  - SWI Prolog (v5.4.2 or above)
    Available from `http://www.swi-prolog.org/`

  See Chapter 9 for details of how to use other Prolog implementations.

Chapter 3

Distribution

The present distribution (‘supple’ is the root) contains the following project directories and files:

- build.xml: the build for ANT necessary to construct the system
- classes: the java classes
- config: contains the configuration files the wrapper needs to translate information from Gate into SUPPLE. Two files can be found here mapping.config and feature_table.config. See Section 7 for an explanation of how the mapping is specified.
- docs: documentation. Contains this document.
- lib: libraries necessary to compile and run different prolog implementations.
- src: the java source files.
- creole.xml the configuration of the Gate SUPPLE Wrapper

Chapter 4

Building the system

You will have to edit the build.xml file and adapt the ‘user modifiable options’ to your particular settings.

- *ant plcafe* constructs the PrologCafe version of the parser
- *ant sicstus* constructs the SICStus version of the parser
- *ant swi* constructts the SWIProlog version of the parser

Chapter 5

Running the parser in a Gate Gui

To run the parser from the Gate GUI you need to load the creole.xml that comes with this distribution, you have to this in the usual way in Gate. If you want a standalone application, just look at the testing code provided in the source. In order to parse a document you will need to construct an application that has:

- document reset (usefull)
- tokeniser (mandatory)
- splitter (mandatory)
- POS-tagger (mandatory)
- Morphology (mandatory)
- Bottom-Up Chart Parser (mandatory) with parameters
  - mapping file (config/mapping.config)
  - feature table file (config/feature_table,mapping)
  - parser file (supple.plcafe, supple.sicstus or supple.swi)
  - the classname of the Prolog wrapper (i.e. shef.nlp.supple.prolog.SICStusProlog)

  You can take a look at build.xml to see examples of invocation for the different implementations.

IMPORTANT NOTE: The wrapper writes temporary files in your temp directory, these files are deleted once the process is finished *unless* the debug option is set to *true* in which case the temporary files will be kept in your file system. Do not forget to turn the debug option to false in order to avoid disk space problems.

Chapter 6

Running the parser standalone

One test for each configuration is provided with the build.xml.

- *ant test.plcafe* tests the PrologCafe implementation
- *ant test.sicstus* tests the Sicstus implementation
- *ant test.swi* tests the SWI Prolog implementation

The program run is shef.nlp.supple.TestSuite which you can use as a base for developing your own application.

Chapter 7

Configuration files

Two files are used to pass information from Gate to the SUPPLE parser: the *mapping* file and the *feature table* file.

7.1 Mapping file

The mapping file specifies how annotations produced using Gate are to be passed to the parser. The file is composed of a number of pairs of lines, the first line in a pair specifies a Gate annotation we want to pass to the parser. It includes the AnnotationSet (or default), the AnnotationType, and a number of features and values that depend on the AnnotationType. The second line of the pair specifies how to encode the Gate annotation in a SUPPLE syntactic category, this line also includes a number of features and values. As an example consider the mapping:

```
Gate;AnnotationType=Token;category=DT;string=&S
SUPPLE;category=dt;m_root=&S;s_form=&S
```

It specifies how a determinant (‘DT’) will be translated into a category ‘dt’ for the parser. The construct ‘&S’ is used to represent a variable that will be instantiated to the appropriate value during the mapping process. More specifically a token like ‘The’ recognised as a DT by the POS-tagging will be mapped into the following category:

```
dt(s_form:‘The’,m_root:‘The’,m_affix:‘_’,text:‘_’).
```

As another example consider the mapping:

```
Gate;AnnotationType=Lookup;majorType=person_first;minorType=female;string=&S
SUPPLE;category=list_np;s_form=&S;ne_tag=person;ne_type=person_first;gender=female
```

It specified that an annotation of type ‘Lookup’ in Gate is mapped into a category ‘list_np’ with specific features and values. More specifically a token like ‘Mary’ identified in Gate as a Lookup will be mapped into the following SUPPLE category:

```
list_np(s_form:‘Mary’,m_root:‘_’,m_affix:‘_’,
text:‘_’,ne_tag:‘person’,ne_type:‘person_first’,gender:‘female’).
```

7.2 Feature table

The feature table file specifies SUPPLE ‘lexical’ categories and its features. As an example an entry in this file is:

```
n;s_form;m_root;m_affix;text;person;number
```

which specifies which features and in which order a noun category should be writen. In this case:

```
n(s_form:...,m_root:...,m_affix:...,text:...,person:...,number:....).
```

Chapter 8

Parser and Grammar

The parser which is a Bottom-up Chart Parser builds a semantic representation compositionally, and a 'best parse' algorithm is applied to each final chart, providing a partial parse if no complete sentence span can be constructed. The parser uses a feature valued grammar. Each `Category` entry has the form:

```
Category(Feature1:Value1,...,FeatureN:ValueN)
```

where the number and type of features is dependent on the category type (see Section 7.2). All categories will have the features `s_form` (surface form) and `m_root` (morphological root); nominal and verbal categories will also have `person` and `number` features; verbal categories will also have `tense` and `vform` features; and adjectival categories will have a `degree` feature. The `list_np` category has the same features as other nominal categories plus `ne_tag` and `ne_type`.

Syntactic rules are specifed in Prolog with the predicate $rule(LHS, RHS)$ where LHS is a syntactic category and RHS is a list of syntactic categories. A rule such as $BNP\_HEAD \Rightarrow N$ ("a basic noun phrase head is composed of a noun") is writen as follows:

```
rule(bnp_head(sem:E^[[R,E],[number,E,N]],number:N),
[n(m_root:R,number:N)]).
```

Where the feature 'sem' is used to construct the semantics while the parser processes input, and E, R, and N are variables tobe instantiated during parsing.

The full grammar of this distribution can be found in the prolog/grammar directory, the file load.pl specifies which grammars are used by the parser. The grammars are compiled when the system is built and the compied version is used for parsing.

8.0.1 Mapping Named Entities

SUPPLE has a prolog grammar which deals with named entities, the only information required is the Lookup annotations produced by Gate, which are specified in the mapping file. However, you may want to pass named entities identified with your own Jape grammars in Gate. This can be done using a special syntactic category provided with this distribution. The category sem_cat is used as a bridge between GATE named entities and the SUPPLE grammar. An example of how to use it (provided in the mapping file) is:

```
Gate;AnnotationType=Date;string=&S
SUPPLE;category=sem_cat;type=Date;text=&S;kind=date;name=&S
```

which maps a named entity 'Date' into a syntactic category 'sem_cat'. A grammar file called semantic_rules.pl is provided to map sem_cat into the appropriate syntactic category expected by the phrasal rules. The following rule for example:

```
rule(ne_np(s_form:F,sem:X^[[name,X,NAME],[KIND,X]]),[
sem_cat(s_form:F,text:TEXT,type:'Date',kind:KIND,name:NAME)]).
```

is used to parse a 'Date' into a named entity in SUPPLE which in turn will be parsed into a noun phrase.

Chapter 9

Using Other Prolog Implementations

You have to write a new Prolog wrapper by extending shef.nlp.supple.prolog.Prolog and you will probably have to write a Prolog file to compile the parser (look at mkparser-*.pl) for examples of how to do this.
